# Supplementary material for: Disruption of tRNA biogenesis enhances proteostatic resilience, improves later-life health, and promotes longevity
Source: PLoS Biol. 2024 Oct 22;22(10):e3002853. doi: 10.1371/journal.pbio.3002853 (PMC11495624; doi:10.1371/journal.pbio.3002853)
Supplement: S1 Table — In each case trial A is presented in Fig 2. (DOCX) [file pbio.3002853.s001.docx]

| **Trial** | **condition** | **Mean Lifespan (days)** | **Extension (%)** | ***N* (dead)** | ***P* value (Log-Rank test)** |  |
| --- | --- | --- | --- | --- | --- | --- |
| A | control (Tm+DMSO) | 9.75 |  | 95 | control (Tm+DMSO) v.s. rpc-1 (Tm+DMSO) | 0.047 |
|  | rpc-1 (Tm+DMSO) | 10.44 | 7.077 | 62 | control (Tm+DMSO) v.s. Control (DMSO only) | 0.0226 |
|  | Control (DMSO) | 10.33 |  | 113 | control (Tm+DMSO) v.s. rpc-1 (DMSO only) | 4.6E-09 |
|  | rpc-1 (DMSO) | 11.48 | 11.133 | 124 |  |  |
| B | control (Tm+DMSO) | 9.57 |  | 93 | control RNAi (Tm+DMSO) v.s. rpc-1 RNAi (Tm+DMSO) | 0.0521 |
|  | rpc-1 (Tm+DMSO) | 10.43 | 8.245 | 76 | control RNAi (Tm+DMSO) v.s. control RNAi (DMSO) | 0.0159 |
|  | Control (DMSO) | 10.67 |  | 66 | control RNAi (DMSO only) v.s. rpc-1 (DMSO) | 0.0043 |
|  | rpc-1 (DMSO) | 11.8 | 9.576 | 100 |  |  |

**Tunicamycin**

**Heatshock**

| **Trial** | **condition** | **Mean Lifespan (days)** | **Extension (%)** | ***N* (dead)** | **P value (Log-Rank test)** |  |
| --- | --- | --- | --- | --- | --- | --- |
| A | control RNAi | 12.40 |  | 159 | control RNAi v.s. rpc-1 RNAi | 0.0025 |
|  | rpc-1 RNAi | 13.42 | 8.225 | 117 | control RNAi v.s. control RNAi (HS) | 0.0006 |
|  | control RNAi (HS) | 11.19 |  | 63 | control RNAi (HS) v.s. rpc-1 RNAi (HS) | 0.0015 |
|  | rpc-1 RNAi (HS) | 12.29 | 9.83 | 184 |  |  |
| B | control RNAi | 12.3 |  | 173 |  |  |
|  | rpc-1 RNAi | 13.4 | 8.943 | 148 | control RNAi v.s. rpc-1 RNAi | 0.0025 |
|  | control RNAi (HS) | 10.7 |  | 59 | control RNAi v.s. control RNAi (HS) | 0.0006 |
|  | rpc-1 RNAi (HS) | 11.9 | 11.21 | 98 | control RNAi (HS) v.s. rpc-1 RNAi (HS) | 0.0015 |

Supplementary Table 1 Demography and statistical analysis of survival with Tunicamycin or after heatshock. In each case trial A is presented in Figure 2.
