# Supplementary material for: Disruption of tRNA biogenesis enhances proteostatic resilience, improves later-life health, and promotes longevity
Source: PLoS Biol. 2024 Oct 22;22(10):e3002853. doi: 10.1371/journal.pbio.3002853 (PMC11495624; doi:10.1371/journal.pbio.3002853)
Supplement: S2 Table — P value is from the log-rank test to RNAi control. For each set of experiments, trial A is presented in S1 Fig. (DOCX) [file pbio.3002853.s002.docx]

| **Trial** | **Strain** | **condition** | **Mean Lifespan (days)** | ***N* (dead)** | **P** |
| --- | --- | --- | --- | --- | --- |
| A | WT | control RNAi + juglone | 3.95 | 101 |  |
|  | WT | *rpc-1* RNAi+ juglone | 4.06 | 94 | 0.654 |
| B | WT | control RNAi + juglone | 3.02 | 112 |  |
|  | WT | *rpc-1* RNAi+ juglone | 3.07 | 91 | 0.4859 |
| C | WT | control RNAi + juglone | 2.99 | 110 |  |
|  | WT | *rpc-1* RNAi+ juglone | 3.11 | 89 | 0.512 |
| A | *hsp-4::GFP* | control RNAi | 11 | 93 |  |
|  | *hsp-4::GFP* | *rpc-1* RNAi | 12.6 | 102 | 1.20E-09 |
|  | *hsp-4::GFP;xbp-1(zc12)* | control RNAi | 11 | 74 |  |
|  | *hsp-4::GFP;xbp-1(zc12)* | *rpc-1* RNAi | 12.1 | 91 | 0.00022 |
| B | *hsp-4::GFP* | control RNAi | 11.47 | 83 |  |
|  | *hsp-4::GFP* | *rpc-1* RNAi | 12.74 | 113 | 0.000012 |
|  | *hsp-4::GFP;xbp-1(zc12)* | control RNAi | 11.21 | 105 |  |
|  | *hsp-4::GFP;xbp-1(zc12)* | rpc-1 RNAi | 12.54 | 107 | 0.0000013 |
| A | WT | control RNAi | 11.01 | 93 |  |
|  | WT | *rpc-1* RNAi | 12.1 | 73 | 0.0033 |
|  | *atf-4(ok576)* | control RNAi | 12.31 | 62 |  |
|  | *atf-4(ok576)* | *rpc-1* RNAi | 13.92 | 100 | 1.5E-08 |
| B | WT | control RNAi | 11.84 | 138 |  |
|  | WT | *rpc-1* RNAi | 12.74 | 142 | 0.0027 |
|  | *atf-4(ok576)* | control RNAi | 13.32 | 50 |  |
|  | *atf-4(ok576)* | rpc-1 RNAi | 12.51 | 74 | 0.0223 |
| C | WT | control RNAi | 11.02 | 90 |  |
|  | WT | *rpc-1* RNAi | 12.12 | 77 | 0.0011 |
|  | *atf-4(ok576)* | control RNAi | 10.75 | 56 |  |
|  | *atf-4(ok576)* | *rpc-1* RNAi | 12.24 | 88 | 0.0005 |
| A | WT | control RNAi | 10.1 | 91 |  |
|  | WT | *rpc-1* RNAi | 11.1 | 126 | 0.0011 |
|  | WT | *atf-6* RNAi | 10.8 | 95 | 0.0569 |
|  | WT | *atf-6* + *rpc-1* RNAi | 11.6 | 117 | 0.0000072 |
|  | WT | control+ *rpc-1* RNAi | 11.1 | 97 | 0.0017 |
|  | WT | control + *atf-6* RNAi | 10.4 | 102 | 0.8908 |
| B | WT | control RNAi | 10.1 | 92 |  |
|  | WT | *rpc-1* RNAi | 11.5 | 136 | 0.00000068 |
|  | WT | *atf-6* RNAi | 10.6 | 98 | 0.0142 |
|  | WT | *atf-6* + *rpc-1* RNAi | 11.6 | 117 | 5.2E-08 |
|  | WT | control+ *rpc-1* RNAi | 11 | 97 | 0.0011 |
|  | WT | control + *atf-6* RNAi | 10.3 | 103 | 0.2819 |
| A | WT | control RNAi | 9.51 | 55 |  |
|  | WT | *rpc-1* RNAi | 10.4 | 126 | 0.0489 |
|  | WT | *dars-1* RNAi | 9.18 | 103 | 0.24 |
|  | WT | *gars-1* RNAi | 9.45 | 75 | 0.89 |
|  | WT | *hars-1* RNAi | 9.73 | 130 | 0.65 |
|  | WT | *yars-1* RNAi | 9.27 | 70 | 0.35 |
| B | WT | control RNAi | 9.41 | 75 |  |
|  | WT | *rpc-1* RNAi | 10.58 | 97 | 0.0049 |
|  | WT | *dars-1* RNAi | 9.23 | 71 | 0.511 |
|  | WT | *gars-1* RNAi | 9.46 | 71 | 0.918 |
|  | WT | *hars-1* RNAi | 9.53 | 55 | 0.680 |
|  | WT | *yars-1* RNAi | 9.21 | 53 | 0.579 |

Supplementary Table 2 Demography and survival analysis for worm juglone resistance and lifespans. P value is from the log-rank test to RNAi control. For each set of experiments, trial A is presented in Supplementary Figure 1.
