# Supplementary figures and images for: Disruption of tRNA biogenesis enhances proteostatic resilience, improves later-life health, and promotes longevity
Source: PLoS Biol. 2024 Oct 22;22(10):e3002853. doi: 10.1371/journal.pbio.3002853 (PMC11495624; doi:10.1371/journal.pbio.3002853)

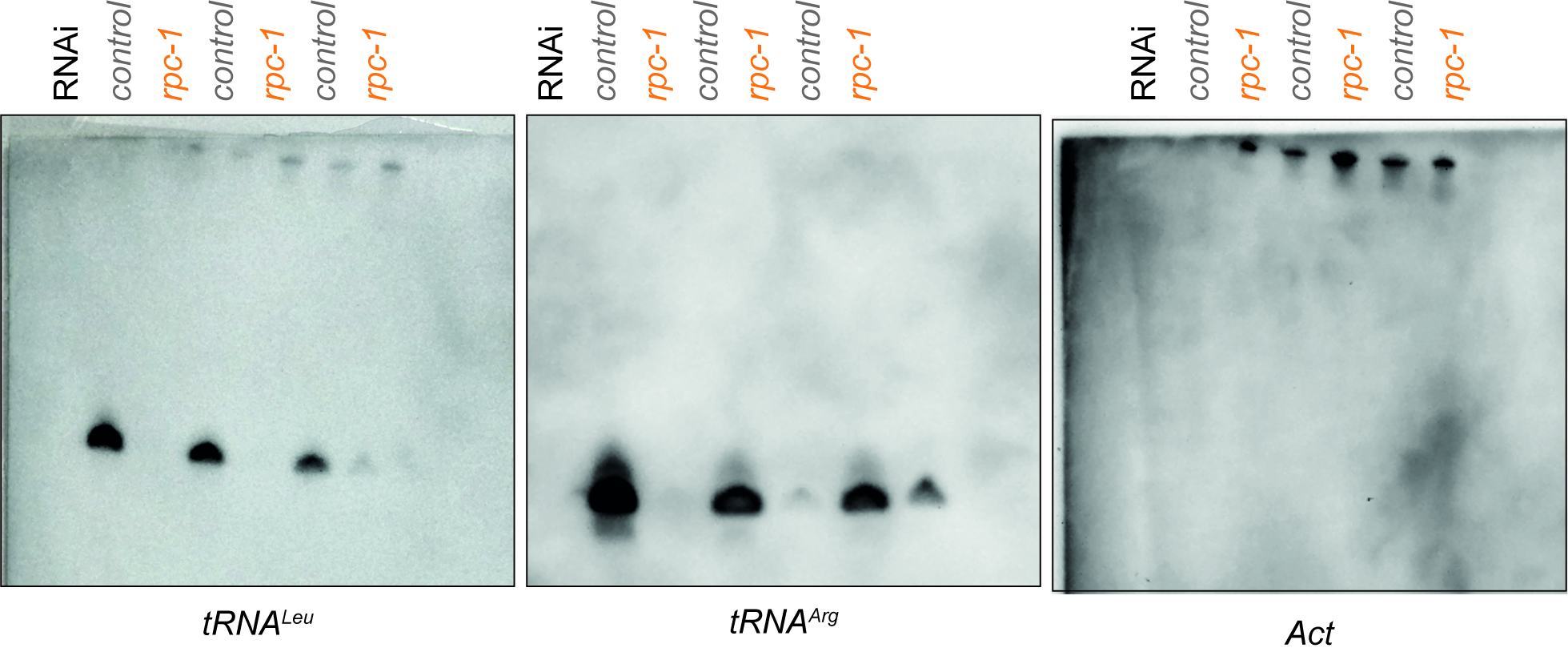

Supplement: S1 Fig — Full images of northern blots presented in Fig 1B. Note that the Actin band corresponding to 1 wild-type sample was accidentally lost from the blot. (TIF) [file pbio.3002853.s003.tif]

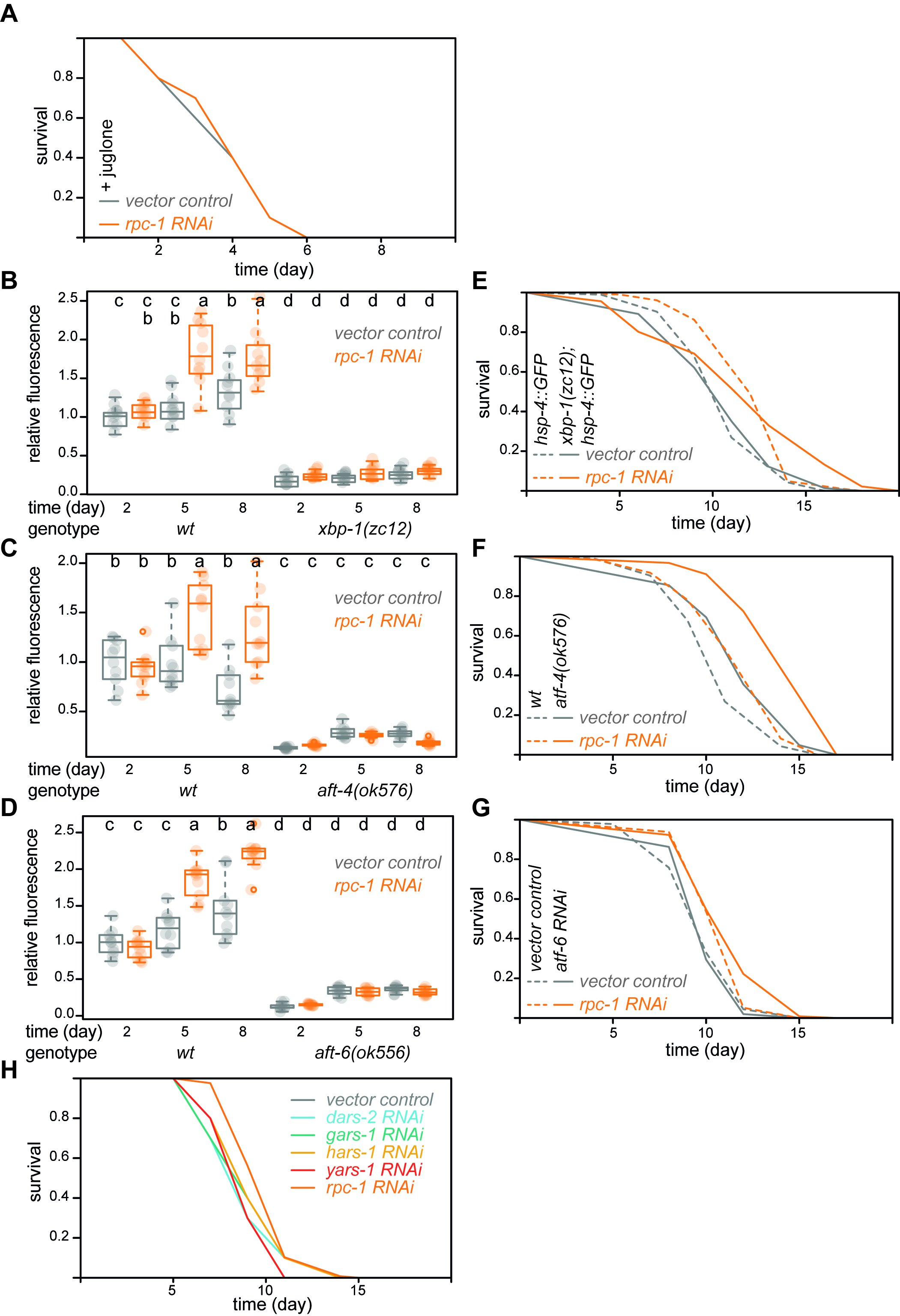

Supplement: S2 Fig — (A) Survival of control or xbp-1 RNAi worms exposed to juglone. (B–D) Expression of the hsp-4::GFP reporter in rpc-1 RNAi- or vector control-treated worms that were either wild type or carried mutations in xbp-1, atf-4 or atf-6. LM analysis with a fully factorial design (age, genotype, RNAi) showed significant effects of all covariates and all interactions (p < 0.05) for all 3 experiments. The letters on top of the graphs indicate the results of the Tukey–Kramer HSD test, where outcomes that are not connected by a letter within an experiment are significantly different (p < 0.05). Boxplots show quantiles with individual data points overlayed. (E, F) Lifespans of control or xbp-1 or atf-4 mutant worms treated with control or rpc-1 RNAi. (G) Lifespans of worms treated with atf-6 RNAi, rpc-1 RNAi, or both. (H) Lifespans of worms treated with rpc-1 RNAi or RNAi against individual aminoacyl tRNA synthases whose knockdown was able to induce hsp-4::GFP. For survival and lifespans, demography, statistics and additional repeats are shown in S2 Table. Data underlying this figure can be found in S1 Data. (TIF) [file pbio.3002853.s004.tif]

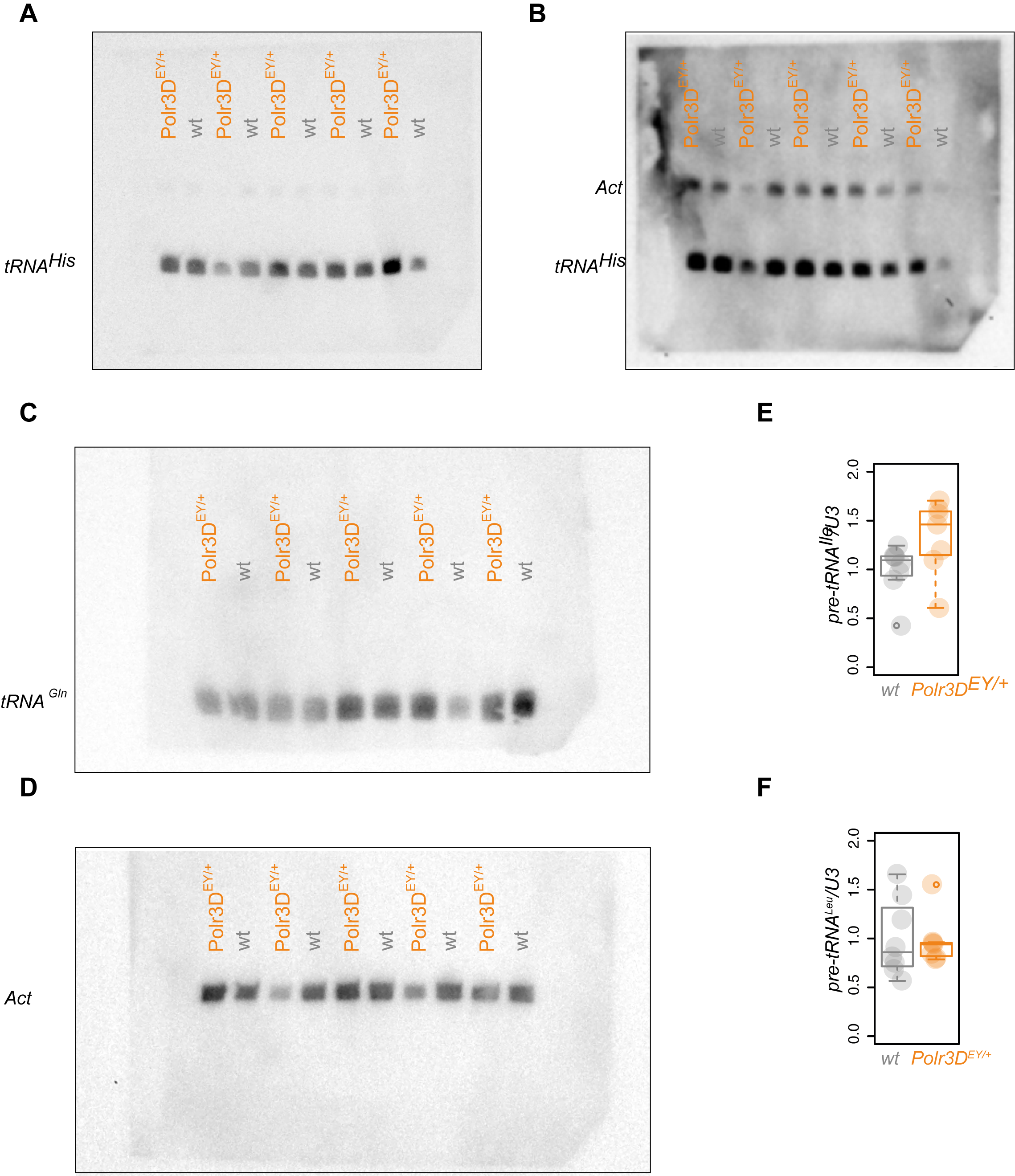

Supplement: S3 Fig — (A and B, C and D) Full images of blots presented and quantified in Fig 3B and 3C. (E) Relative pre-tRNAIle levels (n = 7–8, p = 0.089 t test). (F) Relative pre-tRNALeu levels (n = 7–8, p = 0.88 t test). Boxplots show quantiles with individual data points overlayed. Data underlying this figure can be found in S1 Data. (TIF) [file pbio.3002853.s005.tif]

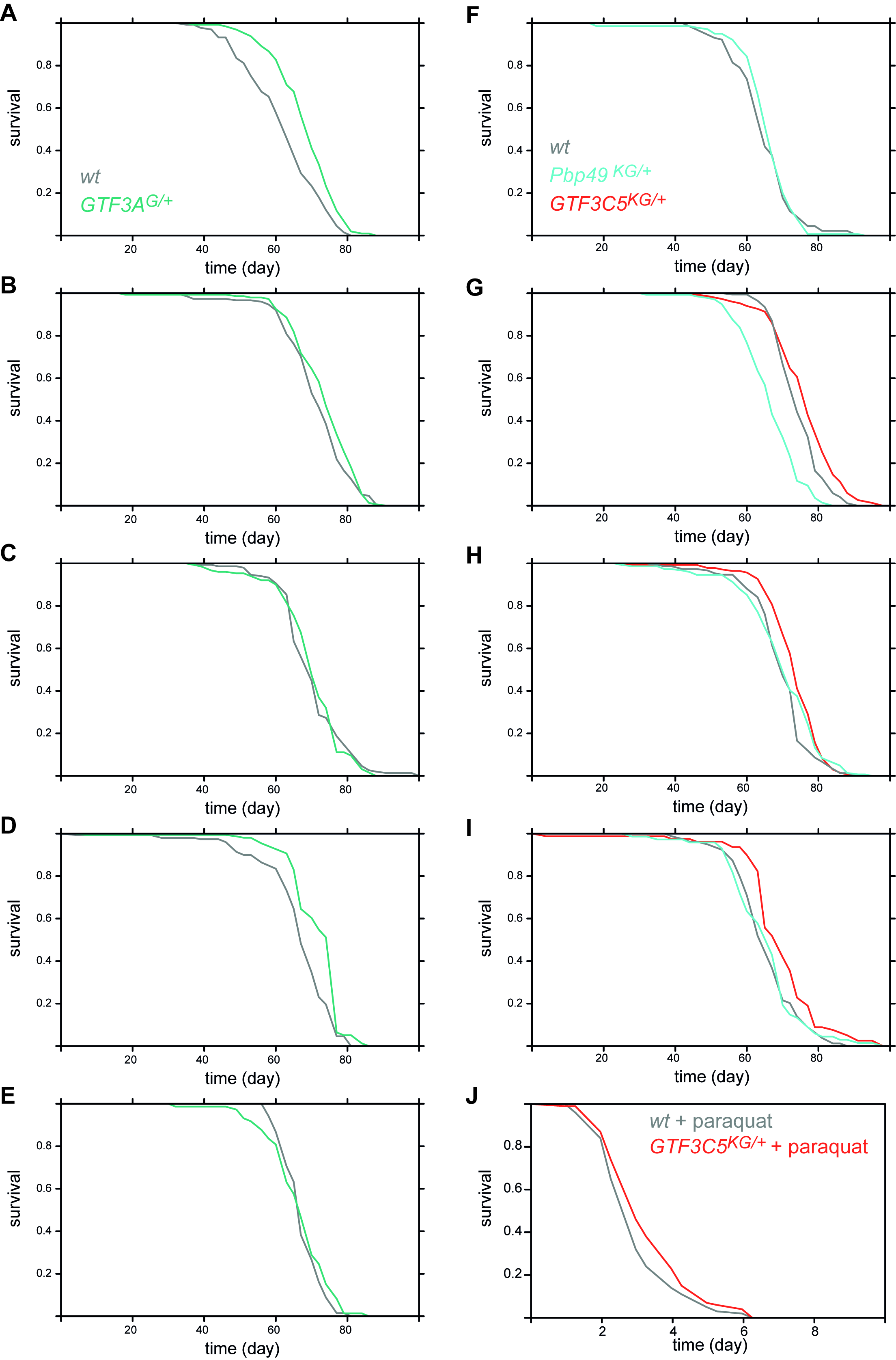

Supplement: S4 Fig — (A) Wild-type n = 133 dead/17 censored flies, GTF3AG/+ n = 119/13, p = 6.6 × 10^−6^. (B) Wild-type n = 64/0, GTF3AG/+ n = 73/1, p = 0.41. (C) Wild type n = 148/2, GTF3AG/+ n = 145/6, p = 0.88. (D) Wild-type n = 109/42, GTF3AG/+ n = 117/38, p = 9.9 × 10^−7^. (E) Wild-type n = 68/0, GTF3AG/+ n = 73/1, p = 0.41. (F) Wild-type n = 124/7, Pbp49KG/+ n = 140/2, p = 0.50. Note this is part of the same experiment as in Fig 4A so the wild-type survival is replotted. (G) Wild-type n = 170/0, Pbp49KG/+ n = 137/0, p = 9.5 × 10^−16^; GTF3C5KG/+ n = 150/0, p = 1 × 10^−3^. (H) Wild-type n = 151/0, Pbp49KG/+ n = 147/2, p = 0.11; GTF3C5KG/+ n = 134/8, p = 9.6 × 10^−4^. (I) Wild-type n = 79/0, Pbp49KG/+ n = 69/3, p = 0.75; GTF3C5KG/+ n = 79/0, p = 2.3 × 10^−3^. (J) Survival of paraquat exposure (wild-type n = 100/0, GTF3AG/+ n = 100/0, p = 0.059). All p values are from log-rank test to wild type. Data underlying this figure can be found in S1 Data. (TIF) [file pbio.3002853.s006.tif]

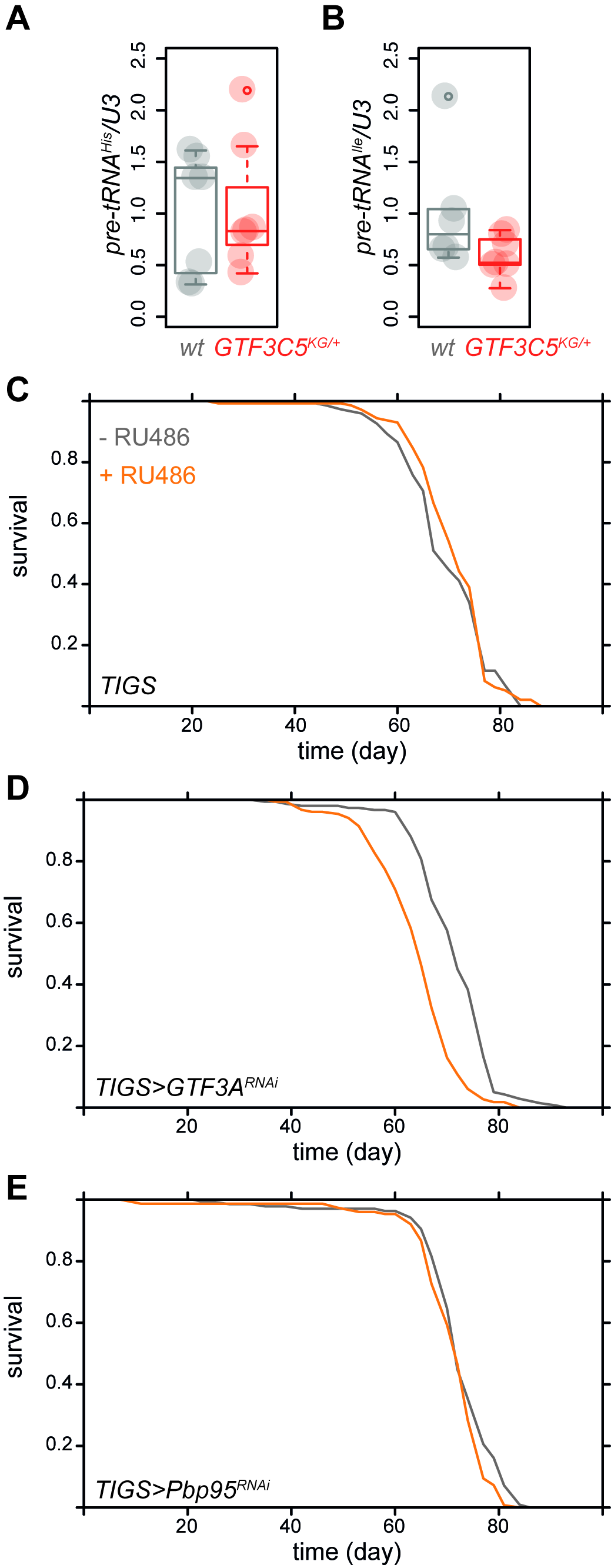

Supplement: S5 Fig — (A) Relative pre-tRNAHis levels (n = 6–7, p = 0.88 t test). (B) Relative pre-tRNAIle levels (n = 7, p = 0.15 t test). (C) Driver alone control (-RU486 n = 125 dead/25 censored flies, +RU486 n = 133/11, p = 0.28, log-rank test). (D) TIGS>GTF3ARNAi females (-RU486 n = 149/2, +RU486 n = 149/3, p = 6.4 × 10^–16^, log-rank test). (E) TIGS>Pbp95RNAi females (-RU486 n = 131/15, +RU486 n = 148/2, p = 0.027, log-rank test). Data underlying this figure can be found in S1 Data. (TIF) [file pbio.3002853.s007.tif]

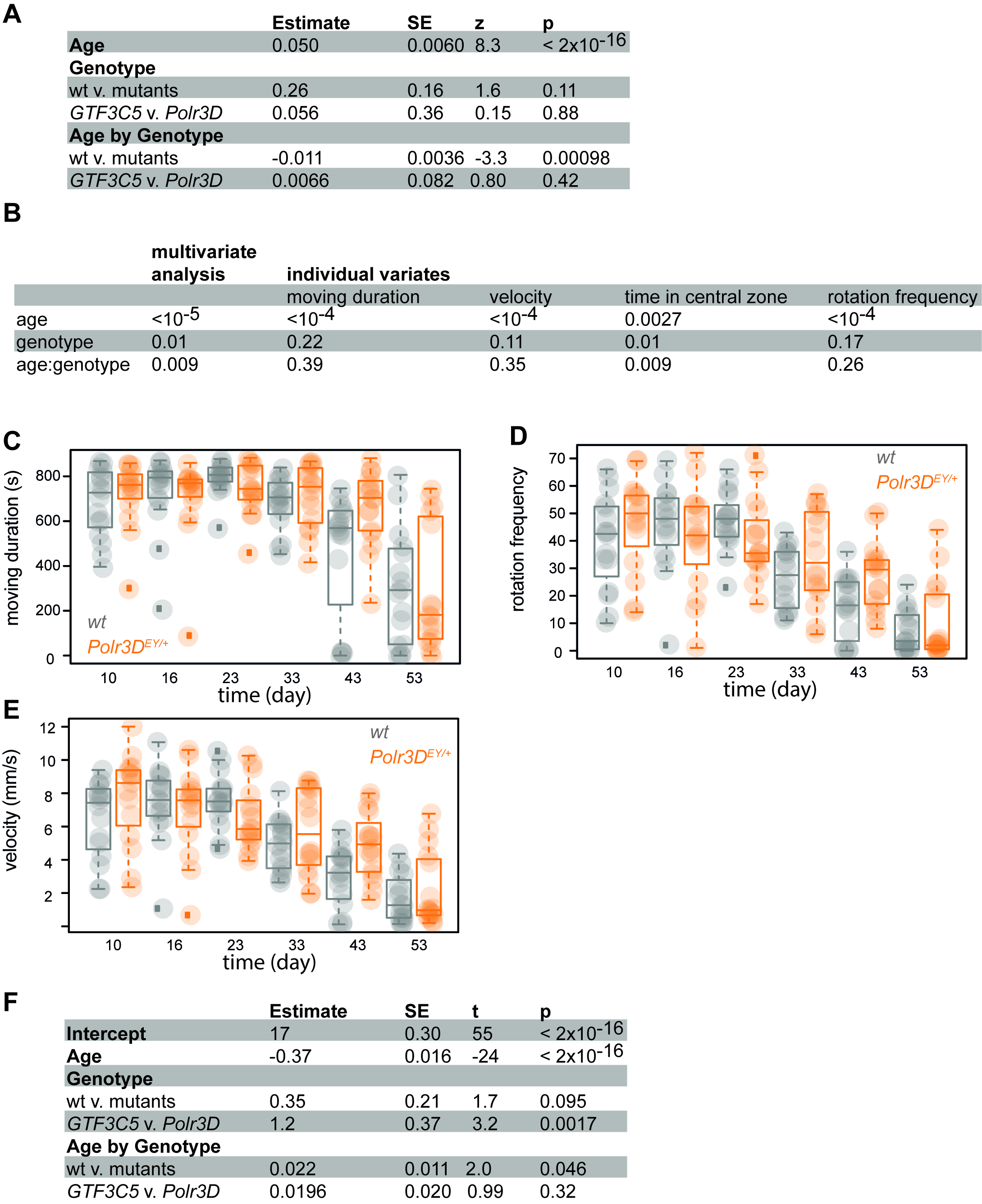

Supplement: S6 Fig — (A) Results of statistical analysis of loss of gut barrier function (smurf assay) on Polr3DEY/+, GTF3C5KG/+ and wild-type females using ordinal logistic regression. (B) P values from the multivariate LM analysis. (C) Moving duration, (D) velocity, and (E) rotation frequency during exploratory walking by Polr3DEY/+ and wild-type females. Boxplots show quantiles with individual data points overlayed. (F) Results of statistical analysis of negative geotaxis assay (climbing assay) on Polr3DEY/+, GTF3C5KG/+ and wild-type females using LM. In all analysis, age is given in days. Data underlying this figure can be found in S1 Data. (TIF) [file pbio.3002853.s008.tif]

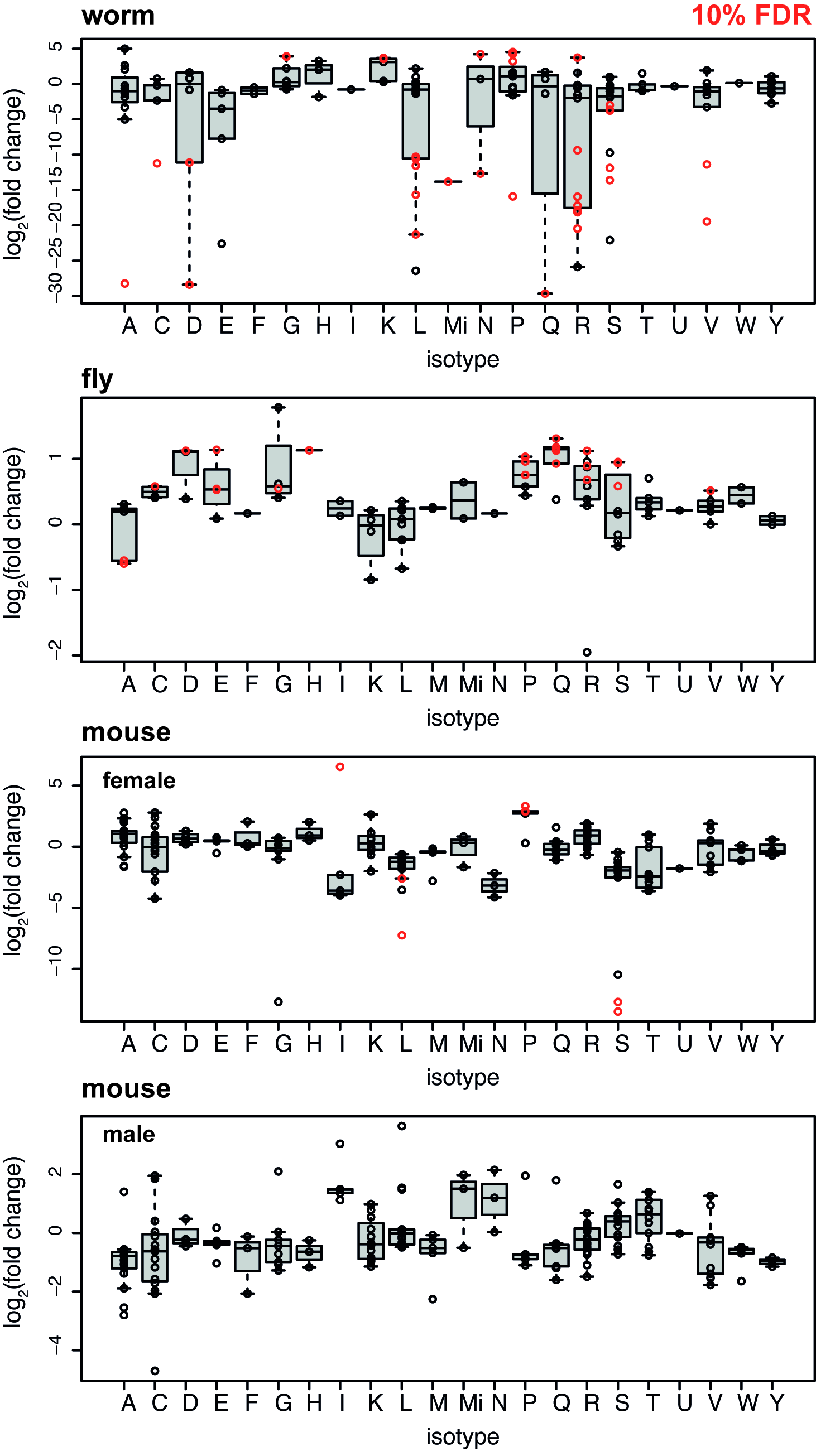

Supplement: S7 Fig — Differential expression (log2 fold change) for tRNAs grouped based on the amino acid they decode in worms, flies (gut), and female and male mice (duodenum) obtained from RNA-Seq analyses. Amino acids are given in single-letter code with Mi indicating initiator tRNAMet, with tRNAs differentially expressed upon loss of function of Pol III in each species indicated in red (10% FDR). Note differences in Y axes between panels. Data underlying this figure can be found in S1 Data. (TIF) [file pbio.3002853.s009.tif]

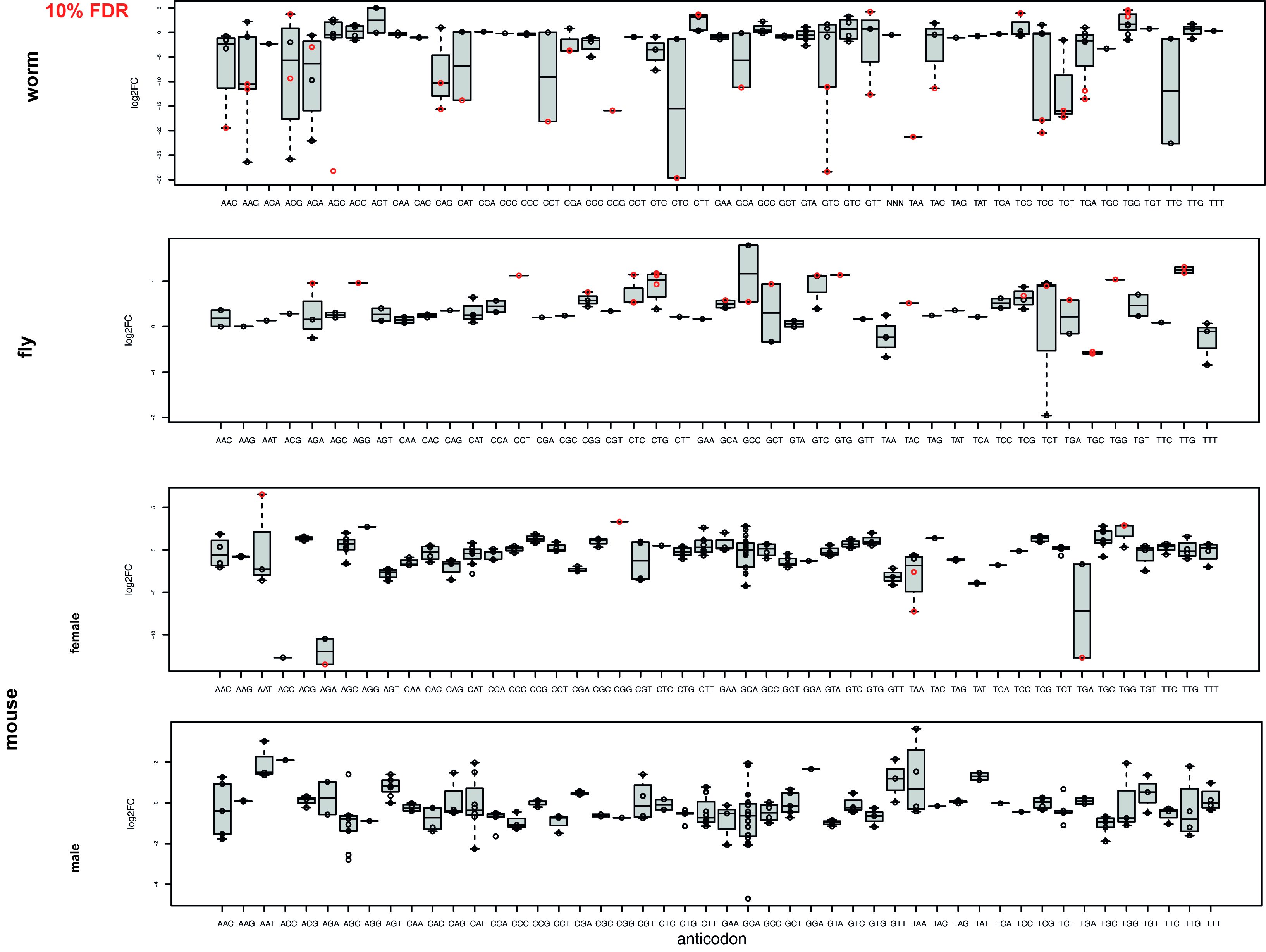

Supplement: S8 Fig — Differential expression (log2 fold change) for tRNAs grouped based on the anticodon in worms, flies (gut), and female and male mice (duodenum) obtained from RNA-Seq analyses. tRNAs differentially expressed upon loss of function of Pol III in each species are indicated in red (10% FDR). Note differences in Y axes between panels. Data underlying this figure can be found in S1 Data. (TIF) [file pbio.3002853.s010.tif]

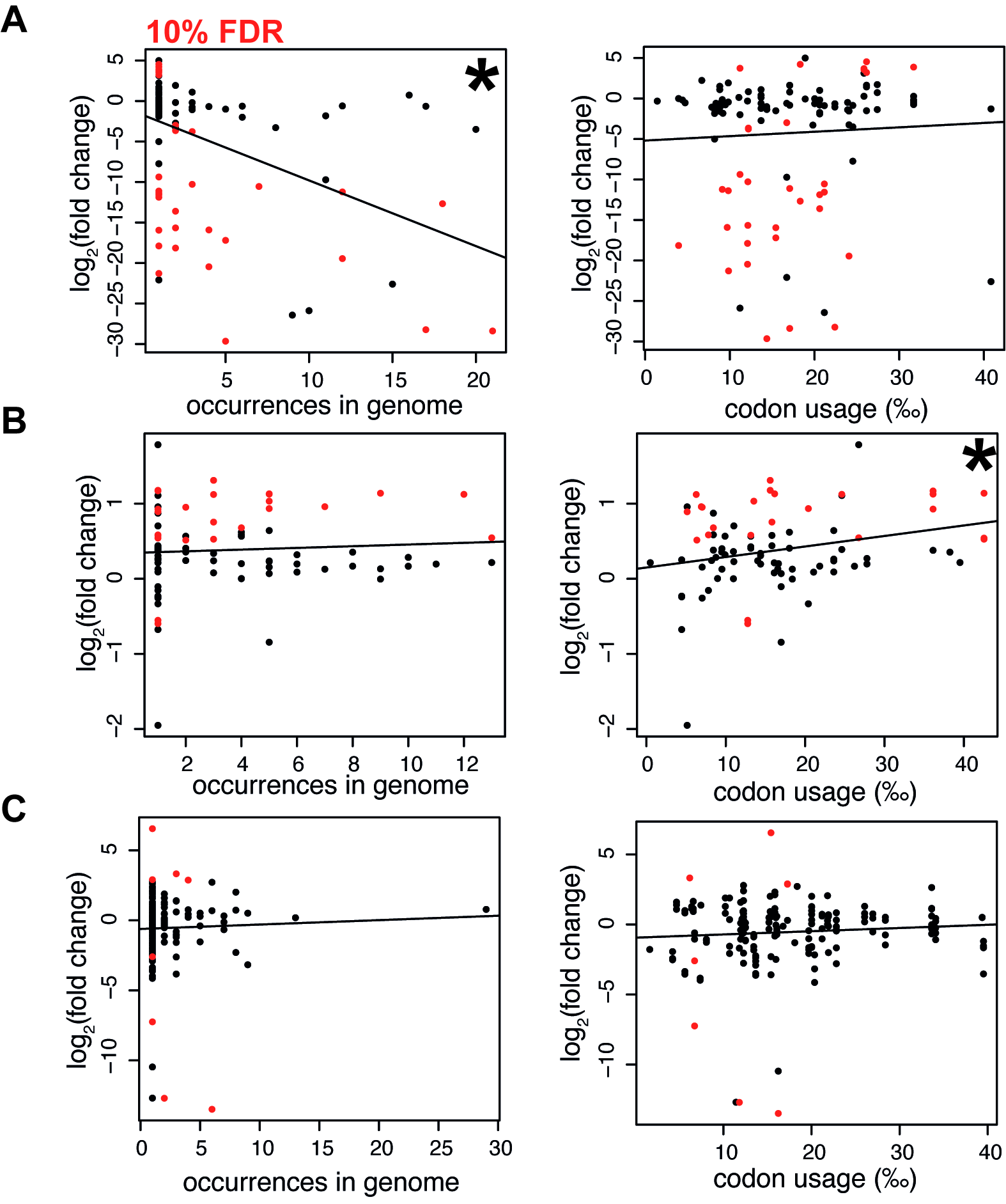

Supplement: S9 Fig — (A) Worms. (B) Flies. (C) Female mice. * Indicates p < 0.05 LM. Data underlying this figure can be found in S1 Data. (TIF) [file pbio.3002853.s011.tif]

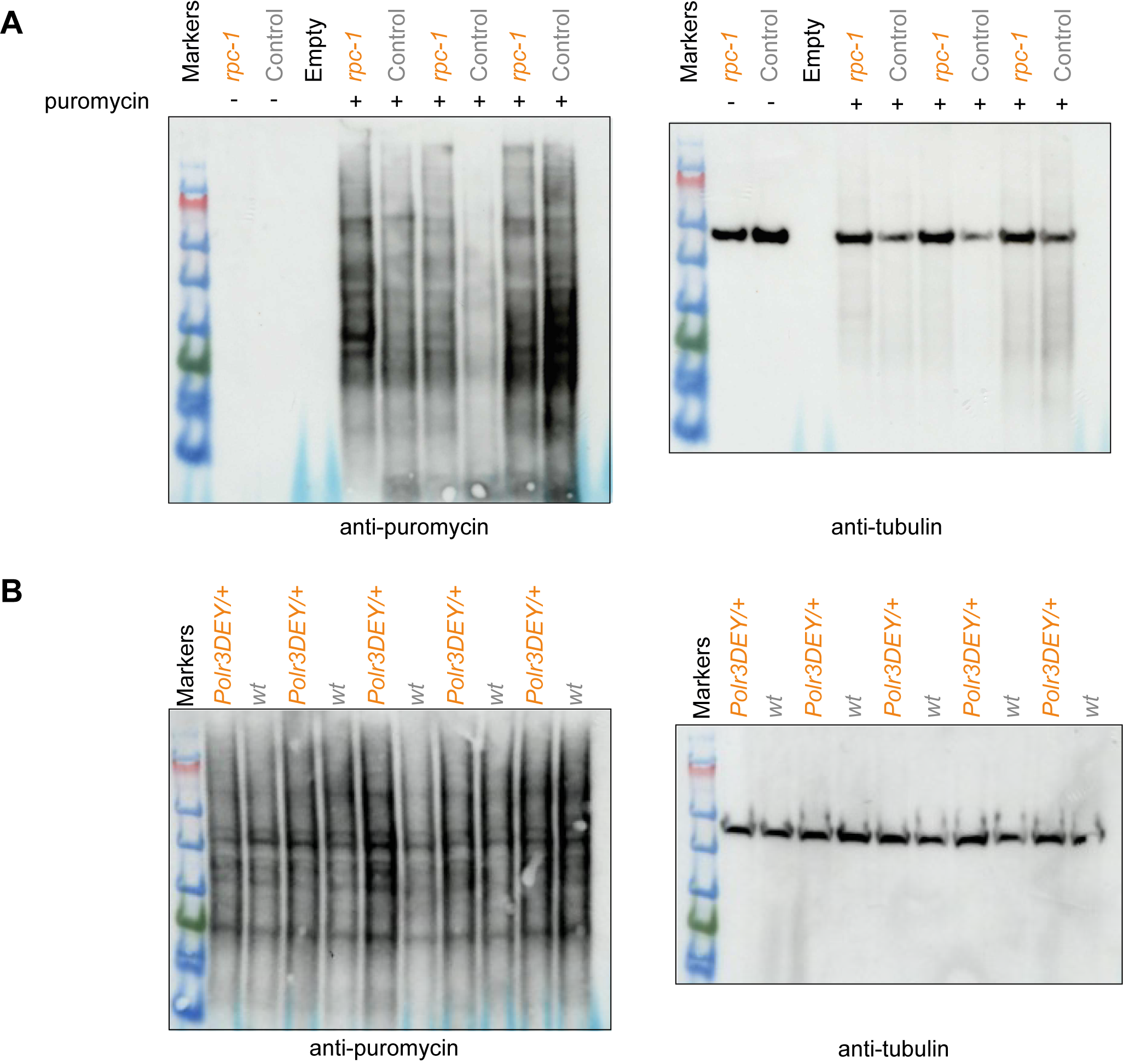

Supplement: S10 Fig — Full blots of quantification of puromycin incorporation in (A) worms and (B) female fly guts. Paired samples that were obtained at the same time are placed next to each other on the blot. Quantifications are given in Fig 7. (TIF) [file pbio.3002853.s012.tif]

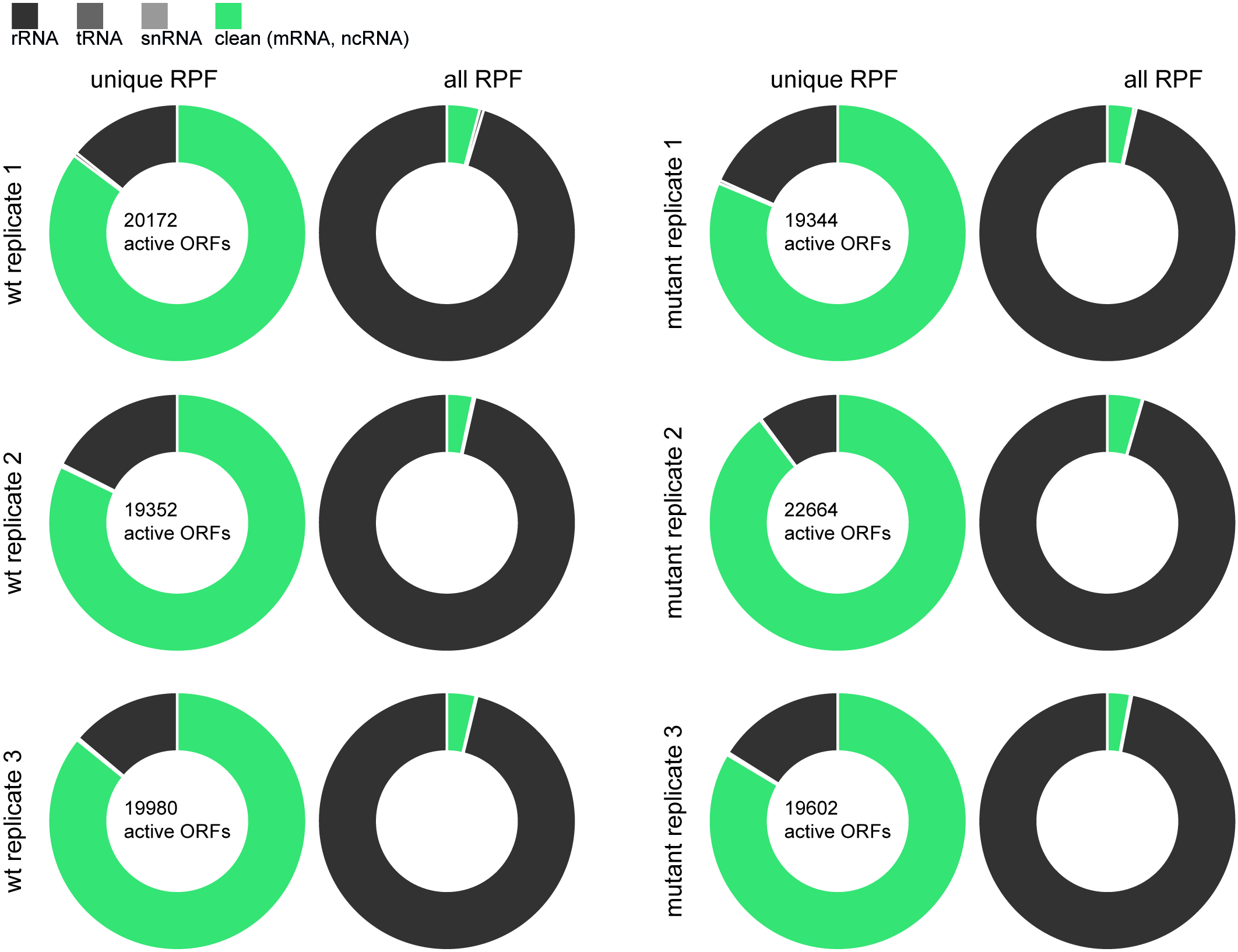

Supplement: S11 Fig — Mappings to types of RNA and open reading frames (ORFs) for unique or all ribosome protected fragments (RPF) in 3three replicate samples of wild-type or mutant (Polr3DEY/+) female flies (whole body). Data underlying this figure can be found in S1 Data. (TIF) [file pbio.3002853.s013.tif]
