# Supplementary material for: Disruption of tRNA biogenesis enhances proteostatic resilience, improves later-life health, and promotes longevity
Source: PLoS Biol. 2024 Oct 22;22(10):e3002853. doi: 10.1371/journal.pbio.3002853 (PMC11495624; doi:10.1371/journal.pbio.3002853)
Supplement: S1 Raw Images — These are also presented in the relevant supplementary figures. (PDF) [file pbio.3002853.s014.pdf]

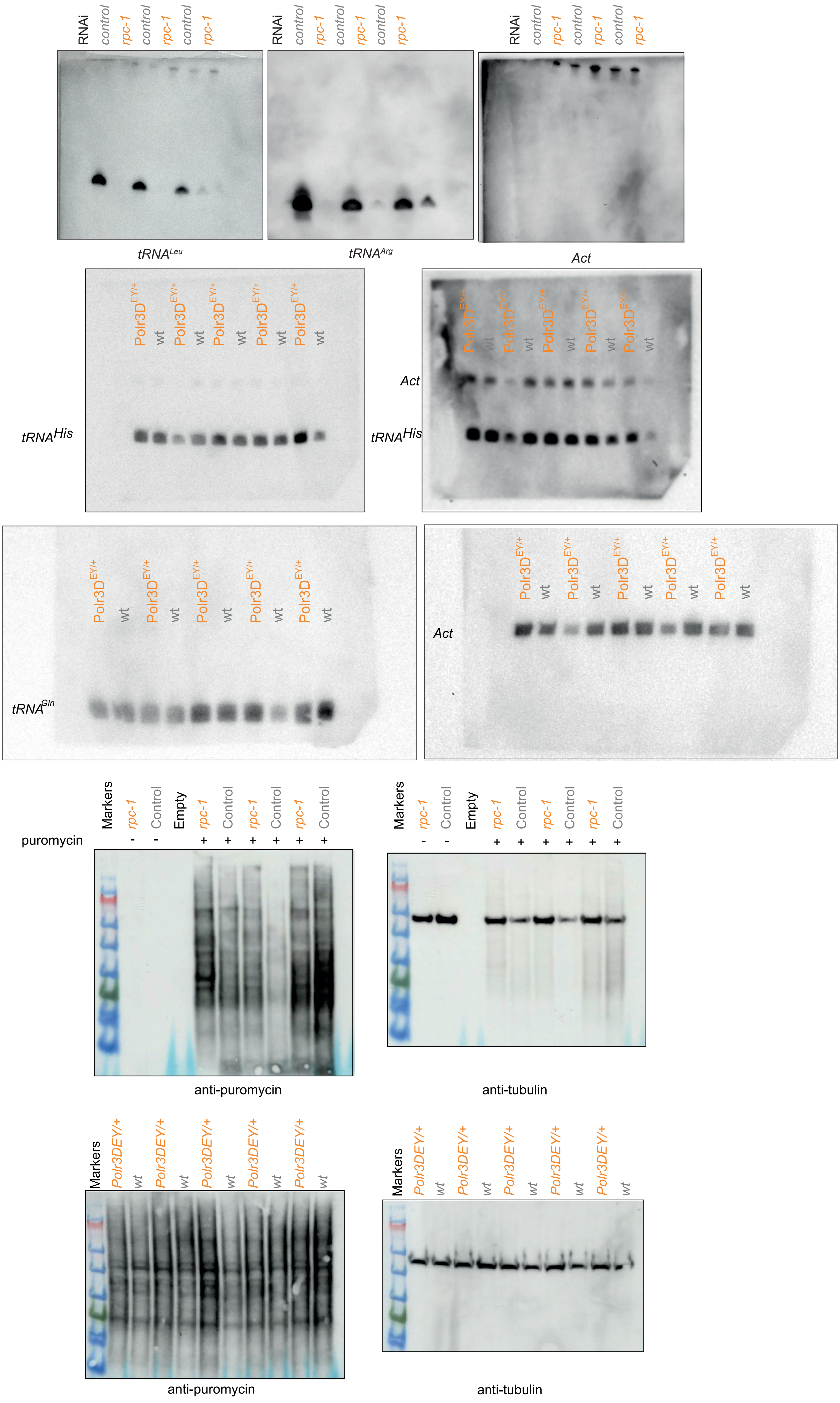
RNAi
control
rpc-1
control
rpc-1
control
rpc-1
tRNALeu
tRNAArg
Act
Polr3DEY/+
wt
tRNAHis
Act
tRNAGln
Markers
rpc-1
Control
Empty
puromycin
-
+
anti-puromycin
anti-tubulin
Polr3DEY/+
wt
anti-puromycin
anti-tubulin
