# Supplementary material for: Disruption of tRNA biogenesis enhances proteostatic resilience, improves later-life health, and promotes longevity
Source: PLoS Biol. 2024 Oct 22;22(10):e3002853. doi: 10.1371/journal.pbio.3002853 (PMC11495624; doi:10.1371/journal.pbio.3002853)
Supplement: Attachment — Submitted filename: Reply_to_reviewer_comments.pdf [file pbio.3002853.s016.pdf]

We are grateful to the reviewers and the academic editor for the time they invested in reviewing our manuscript and for their insightful and constructive comments. We believe we have addressed their concerns in full. In this revised version of the manuscript, we have included numerous new experimental data and analyses, presented in over 25 new figure panels, as well as changed several sections of the text. These are described in the rebuttal below, with major changes also highlighted in the manuscript text of the pdf containing text and all figures.

Comments of the Academic Editor:

1. Can you effectively distinguish "the effect of pol III inhibition on proteostatic resistance from its overall effect on lifespan"? This was raised by Reviewers 1 and 2 and it will be important to address/clarify it.

   We have now added experimental evidence that loss of function in Pol III in worms and flies does not result in resistance to oxidative stress (Supplementary Figures 2A and 4J). Hence, the resistance to proteotoxic stress is specific to that stress and distinguishable from lifespan.

2. Several concerns have been raised regarding the inconsistency in tRNA levels across species under the experimental conditions (e.g. pol III inhibition). An orthogonal method such as northern blotting is needed to confirm a number of the changes in tRNA levels, given that misleading results could plausibly occur from RNA-seq given that tRNAs are highly modified. Although you have completed a demethylation step, this is sufficient and a subset of tRNAs should be chosen for additional confirmation and to verify the differential effects of pol3 inhibition in worms vs. flies. In addition, Reviewer 2 noted "the surprising observation that many tRNAs are upregulated in heterozygous Polr3d mutants" and this result should be verified with an orthogonal method as well.

   We have now verified the RNA-Seq results from worms and flies with northern blots (Figures 1B, 3B and 3C, Supplementary Figures 1, 3A, 3B, 3C and 3D). Additionally, in flies, we have measured pre-tRNA expression with qPCR, as well as through additional analysis of the RNA-Seq data. We observe for $tRNA^{His}$ that the levels of the pre-processed transcript produced by Pol III are decreased while the levels of the mature tRNA are increased (Figures 3D and 3E, Supplementary Figures 3E and 3F). Hence, the likely reason for increased levels of many tRNAs in flies is compensation at the level of mature tRNAs. Please note that the variations we observe between species are not inconsistencies but rather differences between species.

3. It will be "important to provide information about global translation rates in pol III-inhibited vs control organisms or cell lines", with a comparison between worms and flies as a priority, as suggested by Reviewer 3. In addition, this reviewer thinks it is also important assessing translation.

   We now provide measurements of bulk translation in worms and flies (Figure 7D and 7E, Supplementary Figure 10). We now show that these are consistent with our findings on tRNA levels. Additionally, we have performed a full Ribo-

Seq experiments in flies, reporting results that are consistent with our observation of changes in tRNA levels (Figure 7F, Supplementary Figure 11), and providing access to the results of genome-wide analysis (Supplementary data) and well as raw data (deposited in GEO).

Reviewers' comments:

Rev. 1:
This study by Malik et al looks to further explore how inhibition of RNA polymerase III promotes longevity. They focus on the impact of RNA Pol III on disrupting tRNA levels across species including in worms, flies, and mice. They further find that disruption of tRNA synthases is sufficient to induce an unfolded protein response reporter. Furthermore they find that disruption of the TFIIIC transcription factor, which is important for tRNA expression, extends lifespan in flies. Overall I believe this to be an advancement for the field of aging biology by providing new insights into the role tRNAs and RNA Pol III in the aging process.

Major point: The paper primarily focuses on the effects of RNA Pol III disruption being potentially driven through changes in tRNA levels including lifespan extension. While the authors show that disruption of tRNA synthases can induce the UPR, does this also extend lifespan? Was this tested and just not shown? It was shown in yeast and worms that tRNA synthetase inhibitors extend lifespan (Robbins et al 2022), it would further strengthen the authors arguments if specifically disrupting tRNA synthases extended lifespan.

We thank the reviewer for their assessment of our manuscript and their very constructive comments. We have now examined the effects of adult-onset RNAi against a subset of tRNA synthases, individually. These were sufficient to induce UPR but appear insufficient to extend lifespan (Supplementary Figure 2H). This may be because our treatment is not sufficient to cause a substantial enough decrease in tRNA charging, which is supported by the observations of Robbins at al 2022, as well as Rastogi et al 2015 (looking at *vars-2/gpl-4* specifically) and we have added these citations to the text. We have also discussed further conclusions of this experiment in the text and Discussion.

Other points:
Some of the data is not as convincing as the conclusions made.
On page 13 - Similarly, Polr3DEY/+ delayed age-related loss in climbing ability (Figure 5C). The p-value given is (n=99-126, effect of genotype p=4.7x10-6, age p<2x10-16, age by genotype interaction p=2.7x10-6, LM) and yet the data does not look so clear. At 4 and 11 days Polr3D EY/+ appears to have lower climbing ability to the WT. Then only at 18 days the mutant may have slightly higher climbing ability, but this is highly overlapping with WT, and then at 25 days the mutant and WT look essentially identical.

Like humans, our outbred fly population shows substantial individual variation in ageing phenotypes, probably in part due to genetic differences between individual flies. We intentionally use this outbred population as they age in a more biologically relevant/natural way (please see comments by Partridge and Gems, Nature 2007). We display this heterogeneity in our plots by showing data from individual flies. To be

able to quantify the effect of our interventions within this heterogeneity, we employ robust and appropriate statistical analyses that allow us to look at the rate of ageing (age-by-genotype interaction in the regression model) and that are similarly used in human epidemiology. We have now added a short remark to the manuscript text explaining this.

Minor points - its unclear why the authors use shades of yellow-orange and orange-red in some of their graphs, as these colors are sometimes harder to distinguish - Fig 5 and especially Sup Fig 7.

We have tried to use the same colour for the same mutants/conditions across figures. We agree that the colours were difficult to tell apart in Supplementary Figure 7 (now Figure 7A-C) and have now changed them.

Rev. 2: Alessandro Vannini – note that this reviewer has signed his review
In this manuscript, Malik et al show that across species pol III inhibition preferentially alters the levels of a subset of tRNAs, which consistently results in extended lifespan by improving resistance to proteostatic stress. This work is a continuation of the important research done in the authors'laboratories that has uncovered a connection between pol III activity and ageing in several species. While the link between tRNA levels, ageing, and the unfolded protein response (UPR) presented in this manuscript make for an intriguing model, we find the details of these connections to be possibly underexplored.

We thank Dr Vannini for the insightful comments that have substantially helped us improve the manuscript.

In particular, we think that the model proposed in this manuscript could be strengthened by addressing the following points:

- It is not clear whether the effect of pol III inhibition on proteostatic resistance can be effectively distinguished from its overall effect on lifespan. For example, in figure 2A, the shift in survival of rpc-1 RNAi vs vector control worms (orange vs grey solid lines) is interpreted as a sign of increased resistance to tunicamycin. However, rpc1 knockdown generally extends the lifespan of worms and in fact a similar shift can be observed for worms not exposed to tunicamycin (orange vs grey dotted lines). It is therefore difficult to interpret this as a specific effect on resistance to proteostatic stress. It would be useful if the authors could show the survival data from tunicamycin-treated samples/organisms normalised by their non-tunicamycin-treated counterparts. The same applies to figure 2B and figure 4D.

The nature of survival data means that they are not easy to normalise as suggested (they contain censored events are hence require specific statistical analyses). Instead, we chose to address this comment in a different way, by focusing on specificity of the resistance to proteostatic stress. We now show that both in worms and flies, the loss of function in Pol III does not increase their resistance to an agent causing oxidative stress (Supplementary Figures 2A and 4J). This indicates that the resistance to

proteotoxic stress is at least somewhat specific and not the result of a general increase in lifespan.

- Given that there doesn't seem to be any consistency in how specific tRNAs are affected by pol III inhibition across species, and that other mechanisms that affect translation rates are also known to prolong longevity, the authors propose that the effects of tRNA levels on ageing are likely mediated by their effect on translation rates. While we find this hypothesis convincing (generally lower translation rates could indeed explain both the extension in life span and the increase in tolerance of proteostatic stress), it would be important to provide information about global translation rates in pol III-inhibited vs control organisms or cell lines. In particular, it would be interesting to compare differences in translation rates in worms, where pol III inhibition mostly causes a downregulation of tRNA levels, to those of flies, where surprisingly the levels of most tRNA seem to be increased upon pol III inhibition. Are global translation rates affected similarly in both cases, despite levels of tRNAs changing in opposite directions? If this is not the case, how can almost opposite effects on tRNA levels be linked to the same downstream outcome on lifespan?

We have now examined the bulk translation rates in worms and fly guts by assessing puromycin incorporation. Consistent with the observations of tRNA levels, we find that bulk translation is reduced in worms (Figure 7D, Supplementary Figure 10A) while it appears unaffected in flies (Figure 7E, Supplementary Figure 10B). While bulk translation was not reduced in flies, there was still the possibility that translation of specific protein was impacted (due to changes in proportion of tRNAs in the tRNA pool). As a proof of principle, we have now performed Ribo-Seq in flies and indeed find evidence for extensive changes in the rates of translation of individual mRNAs (Figure 7F, Supplementary Figure 11; full analysis in Supplementary Data with raw data deposited to GEO). Hence, even though bulk translation is unaffected in fly guts, there is a likely disruption to translation of specific mRNAs. We have now emphasised in the manuscript text that we think these disruptions to translation (observed as a reduction in bulk translation in worms and as a differential mRNA translation in flies) are the likely triggers for both resistance to proteostatic stress and longevity. Note that even though the disruptions are not identical they appear to cause the same physiological outcome likely as they both affect translation.

- Again related to the surprising observation that many tRNAs are upregulated in heterozygous Polr3d mutants (Fig 3A), it is not clear to us how the results from these organisms, namely the increased expression of BiP (Fig 3B), relate to the data from flies in which recruitment to type II promoters is directly affected (Fig 4). Are tRNA levels also counterintuitively upregulated in GTF3C5 mutants? If they are, as expected, mostly downregulated, this would effectively uncouple the results from figures 3 and 4.

Indeed, the observation of increased levels of tRNAs was surprising. We have now examined further what is happening in the fly gut and obtained an important clue: levels of *pre-tRNA*^{His} are reduced while the levels of the mature *tRNA*^{His} are increased in Pol III loss-of-functin fly guts. This difference between the effects on pre-tRNA and mature tRNA levels is consistent across different methods (RNA-Seq, northern blotting and qPCR; Figures 3B, C, D and E, Supplementary Figures 3C and D). We believe this indicates a reduction in Pol III initiation on at least some type-2 promoters with a

concomitant, compensatory stabilisation of some mature tRNAs. This compensation is likely more extensive in flies than worms as the flies carried the mutant allele though development.
Using qPCR we have now shown that the TFIIIC subunit mutant also has reduced levels of at least one pre-tRNA (out of three examined), namely pre-$tRNA^{Leu}$ (Figure 4E), indicating impaired initiation on at least some type-2 promoters. We believe this essentially couples the results observed in Pol III and TFIIIC mutants.
We do not see significant differences in pre-$tRNA^{His}$ in the TFIIIC mutant, and conversely, we do not see significant differences in pre-$tRNA^{Leu}$ in the Pol III mutant (and neither shows any significant differences in pre-$tRNA^{Ile}$; Supplementary Figures 3E, 3F, 5A and 5B). We think the observed reduction in different pre-tRNAs in Pol III or TFIIIC mutant is plausible - different components of the Pol III machinery may be limiting for initiation on different genes *in vivo*. Furthermore, this is consistent with our conclusion that not all genes are equally affected by the loss of Pol III, not even all tRNA genes.

- In supplementary figure 1 A-C, the authors report the effects of rpc-1 knockdown in wild type worms and worms carrying mutations in each of the three UPR branches. The plots show that in all mutants the general levels of expression are the reporter genes are much lower than in the wild type, independently of the life stage or RNAi treatment. This experiment does not then indicate that all three branches of the UPR are engaged following Pol III inhibition, as the authors conclude, but that mutations in each of the branches are sufficient to completely hinder expression of the reporter gene. It would be useful if the authors could perform similar experiments using a different, possibly endogenous, read-out of the UPR, which is not completely impaired by each of the mutations.

We have now removed these comments and changed the discussion of these results in the manuscript. We also note that this reporter has been excessively used to characterise the UPR in worms *in vivo* and we do not believe we could find a better alternative.

- In the discussion, the authors state that the variation seen in the effect of Pol III inhibition on different tRNAs might be due, among other things, to a difference in tRNA gene copy numbers. This is an interesting hypothesis that could be tested with just some additional analysis of the data presented in this manuscript, by checking if in each species tRNA genes with higher copy numbers are less affected by pol III inhibition and/or vice versa.

We have now examined this. We do find that in worms tRNAs with a higher copy number are more likely to be less abundant in the Pol III loss-of-functions condition; but we find no relationship between copy number and abundance in flies. In flies, we find a relationship between codon usage and abundance, which we do not find in worms. Overall, we think this is again indicating that different forces shape the final tRNA abundance in worms, where the loss-of-function is acute, and flies, where the loss-of-function is present throughout development. The analyses are presented in Supplementary Figure 9.

Rev. 3:
This article by Malik et al. uses several model organisms to better define how pol3 activity is linked to longevity. Interestingly, they observe that tRNAs likely underpin the complex physiological effects, at least in part through activation of the UPR. The unifying theme across model organisms adds credibility to the hypothesis, although the specific effects on tRNAs are distinct in flies and mice. Although female-specific changes are noted, I think the article would benefit from further discussion and speculation about the sex-specific differences. It would be important in future work to determine how translation is affected and whether specific sets of transcripts are sensitive to the tRNA changes. The authors allude to this possibility but addressing this question in a rigorous way might be beyond the scope of this paper.

We thank the reviewer for their supportive comments. We have somewhat expanded the discussion of sexual dimorphism in the section describing the mouse data. Importantly, we now provide measurements of bulk translation in worms and flies (Figure 7D and 7E, Supplementary Figure 10), and these are consistent with our findings on tRNA levels. Additionally, we have performed a full Ribo-Seq experiment in flies, reporting results that are consistent with our observation of changes in tRNA levels (Figure 7F, Supplementary Figure 11), and providing access to the results of genome-wide analysis (Supplementary data) and well as raw data (deposited in GEO). Overall, we now provide evidence that translation is impacted in worms and flies with a loss-of-function in Pol III.

Minor note: In the Figure 2 title, "metabolisms" should be replaced with "metabolism"

We have corrected this error – thank you.
